# Mapping Key Populations to Develop Improved HIV and AIDS Interventions: Multiphase Cross-Sectional Observational Mapping Study Using a District and City Approach

**DOI:** 10.2196/56820

**Published:** 2025-01-30

**Authors:** Pande Putu Januraga, Endang Lukitosari, Lanny Luhukay, Rizky Hasby, Aang Sutrisna

**Affiliations:** 1 Center for Public Health Innovation Faculty of Medicine Udayana University Denpasar Indonesia; 2 Ministry of Health of Indonesia Jakarta Indonesia; 3 Monitoring, Evaluation, and Learning Platform USAID Jakarta Indonesia

**Keywords:** Indonesia, key population, mapping, pandemic, HIV, AIDS, hotspot

## Abstract

**Background:**

Indonesia’s vast archipelago and substantial population size present unique challenges in addressing its multifaceted HIV epidemic, with 90% of its 514 districts and cities reporting cases. Identifying key populations (KPs) is essential for effectively targeting interventions and allocating resources to address the changing dynamics of the epidemic.

**Objective:**

We examine the 2022 mapping of Indonesia’s KPs to develop improved HIV and AIDS interventions.

**Methods:**

In 2022, a district-based mapping of KPs was conducted across 201 districts and cities chosen for their HIV program intensity. This multiphase process included participatory workshops for hotspot identification, followed by direct hotspot observation, then followed by a second direct observation in selected hotspots for quality control. Data from 49,346 informants (KPs) were collected and analyzed. The results from individual hotspots were aggregated at the district or city level, and a formula was used to estimate the population size.

**Results:**

The mapping initiative identified 18,339 hotspots across 201 districts and cities, revealing substantial disparities in hotspot distribution. Of the 18,339 hotspots, 16,964 (92.5%) were observed, of which 1822 (10.74%) underwent a second review to enhance data accuracy. The findings mostly aligned with local stakeholders’ estimates, but showed a lower median. Interviews indicated a shift in KP dynamics, with a median decline in hotspot attendance since the pandemic, and there was notable variation in mapping results across district categories. In “comprehensive” areas, the average results for men who have sex with men (MSM), people who inject drugs, transgender women, and female sex workers (FSWs) were 1008 (median 694, IQR 317-1367), 224 (median 114, IQR 59-202), 196 (median 167, IQR 81-265), and 775 (median 573, IQR 352-1131), respectively. “Medium” areas had lower averages: MSM at 381 (median 199, IQR 91-454), people who inject drugs at 51 (median 54, IQR 15-63), transgender women at 101 (median 55, IQR 29-127), and FSWs at 304 (median 231, IQR 118-425). “Basic” areas showed the lowest averages: MSM at 161 (median 73, IQR 49-285), people who inject drugs at 7 (median 7, IQR 7-7), transgender women at 59 (median 26, IQR 12-60), and FSWs at 161 (median 131, IQR 59-188). Comparisons with ongoing outreach programs revealed substantial differences: the mapped MSM population was >50% lower than program coverage; the estimates for people who inject drugs were twice as high as the program coverage.

**Conclusions:**

The mapping results highlight significant variations in hotspots and KPs across districts and cities and underscore the necessity of adaptive HIV prevention strategies. The findings informed programmatic decisions, such as reallocating resources to underserved districts and recalibrating outreach strategies to better match KP dynamics. Developing strategies beyond identified hotspots, integrating mapping data into planning, and adopting a longitudinal approach to understand KP behavior over time are critical for effective HIV and AIDS prevention and control.

## Introduction

### Background

Indonesia faces a multifaceted HIV epidemic shaped by its extensive archipelago and significant population size: 90% of its 514 districts and cities, home to >260 million people, have reported HIV and AIDS cases [1]. This vast distribution presents unique challenges in the fight against HIV. It is projected that in 2023, there will be 543,509 people living with HIV aged ≥15 years, and the number of new HIV infections will reach 23,040 in that same year (Ministry of Health of Indonesia, unpublished data, 2023).

Although HIV transmission has been reported throughout the nation, the epidemic remains predominantly concentrated among key populations (KPs), including men who have sex with men (MSM), transgender women, female sex workers (FSWs), and people who inject drugs [2]. However, the dynamics of the HIV epidemic differ among these groups. The MSM group has seen a rise in HIV prevalence, in stark contrast to the other groups that have experienced a decline. The most significant reduction in HIV cases was observed in the FSW group: in 2007, the HIV prevalence was 10%, but this figure fell to 2.1% in 2018-2019. A similar decline was noted among the group of people who inject drugs: approximately 52.4% of HIV cases in 2017 decreased dramatically by almost 60% to 13.6% in 2018-2019. Transgender women experienced a 50% reduction in HIV prevalence—from 24.33% in 2007 to 12% in 2018. Conversely, the MSM group exhibited an upward trend: HIV prevalence surged from 5.33% in 2007 to 17.9% in 2018-2019 [3].

The observed decrease in HIV prevalence among KPs—except for the MSM group—is influenced by multiple factors and should not be interpreted as a significant reduction in risk or transmission at the community level. It is crucial to emphasize that the primary transmission mode in new cases reported in 2021 predominantly involved heterosexual groups, specifically transmission from husbands to wives or long-term partners. This includes long-term partners from among KPs. In addition, there remains a concerning deficiency in the HIV care cascade in Indonesia [4]: in 2022, only 42% of people living with HIV were on antiretroviral therapy (ART; Ministry of Health of Indonesia, unpublished data, 2023).

To effectively combat the HIV epidemic, it is paramount that HIV prevention and control initiatives prioritize KPs in Indonesia [4]. Given the country’s unique characteristics, these programs must have precise data about their target populations. KPs were mapped to offer insights into these target groups’ size and role in Indonesia’s HIV dynamics. The most recent mapping was conducted in 2022. What distinguishes the mapping efforts of 2022 is their execution in the post–COVID-19 pandemic period, a time when the pandemic has profoundly altered the public health landscape [5]. Various Indonesian studies have documented shifts in health care behaviors and access among KPs due to the pandemic. Notably, the COVID-19 era has also driven significant changes in digital technology use, impacting both risky behaviors and access to health services among these populations [6,7]. The mapping conducted during this period considers these pandemic-induced changes, offering insights into the evolving dynamics of health service use and risk behaviors in a world altered by the COVID-19 pandemic. This makes the recent mapping efforts particularly relevant and timely, reflecting the new normal of health care engagement and the needs of KPs in Indonesia. Notably, the findings from this mapping exercise have not been reported in scientific publications.

### Objectives

This study examines the outcomes of district- and city-level KP mapping in Indonesia. First, it aims to pinpoint hotspots for KPs, describing the types of places where these individuals gather, meet sexual partners, or obtain drugs for injection. In addition, the study seeks to estimate the size of KPs in selected districts and cities. Finally, it aims to compare the 2022 mapping data for 201 districts and cities with data from the pre–COVID-19 period, shedding light on the changes and continuities in KP distributions influenced by the pandemic’s impact on public health and societal norms.

## Methods

### Mapping Frameworks

The 2022 mapping followed the basic methodology of the Priorities for Local AIDS Control Efforts (PLACE) strategy. PLACE is a cross-sectional data collection approach that recruited informants directly from hotspot locations. This method is a location based and aims to identify specific areas where targeted outreach services could effectively reach populations at high risk of HIV infection [8,9].

The core objective of the 2022 mapping effort using the PLACE methodology was to identify and map locations frequented by individuals engaged in high-risk behaviors, such as meeting sexual partners or obtaining drugs for injection. Once these locations were mapped, informants were selected and recruited from these areas, especially if the number of locations exceeded a predetermined maximum quota. Therefore, PLACE was a comprehensive strategy that involved not only identifying hotspot locations but also extensively mapping these areas. Subsequently, informants from the selected locations were systematically recruited and interviewed.

### Definitions

For the purpose of mapping to identify KP sizes in Indonesia, standard definitions of the target groups were needed. FSWs were defined as women who sold sex for money or goods as their primary source of income. These women worked in brothels, streets, or public places where customers came to buy sex. They may or may not have worked for a manager or pimp. This group also included women who worked in entertainment venues (such as karaoke, bars, massage parlors, etc) and sold sex to the customers encountered in these venues. Sex transactions occurred both within entertainment venues and outside, and the owner or manager of these venues may or may not have facilitated such transactions.

MSM, defined as men who have sex with their male partners, included those who identified as gay, bisexual, or straight, as well as individuals who sold and bought sex from other men (male sex workers). Transgender women included biologically male individuals who identified as female and behaved and dressed like women. People who inject drugs comprised men and women who had injected drugs in the past 12 months, excluding those prescribed by a medical professional. This group also included individuals receiving opiate substitution therapy or participating in abstinence-based programs.

The time variable of the past 12 months was included in the definition of people who inject drugs to ensure accurate and current data on drug injection behaviors, which could fluctuate more frequently and were subject to recent intervention impacts. By contrast, behaviors and identities within the populations of FSWs, MSM, and transgender women were generally more stable over time and did not typically require a recent time frame for accurate classification.

### Mapping Procedures

The mapping procedure was carried out in multiple phases, beginning with the selection of districts and cities. For the 2022 KP mapping, 201 districts and cities were chosen proportionally based on a classification system established by the Ministry of Health, which categorized regions into 3 levels of HIV control programs: “basic,” “medium,” and “comprehensive.” Basic regions had minimal HIV testing and treatment services; medium regions provided additional services, including health promotion, HIV prevention, outreach, and condom distribution; and comprehensive regions offered a full range of HIV and AIDS services, including all levels of prevention and advocacy related to health laws in HIV control.

The selection criteria included various parameters, such as the estimated population numbers for MSM, transgender women, FSWs, and people who inject drugs as of 2020. Additional factors considered included the cumulative number of new HIV cases reported from 2011 to 2021, projected new cases for the period from 2022 to 2024, and the number of people living with HIV who were receiving ART in 2021.

The comprehensive category consisted of 100 districts and cities, accounting for 80.59% (42,681/52,955) of the total new HIV cases, individuals receiving ART, and most KPs. Of these 100 districts and cities, 97 (97%) were selected as mapping locations in 2022. The medium category included 138 districts and cities with a significant proportion of new cases and KPs, with 89 (64.5%) selected for mapping in 2022. The basic category comprised 276 districts and cities that had limited interventions in HIV testing services and ART, of which 15 (5.4%) were included in the 2022 mapping. The number of districts and cities selected for KP mapping in 2022 was proportional to the estimated number of KPs in 2020, as well as the district and city categories for HIV control programs. In addition, districts and cities that participated in the integrated biological and behavioral survey of KPs in 2023 were confirmed as locations for mapping.

Overall, the mapping activities covered 201 (39.1%) of the 514 districts and cities in Indonesia. Identifying hotspots within the 201 selected districts and cities was achieved through 1-day participatory workshops held in July and August 2022. These workshops included 4 to 13 stakeholders from each district or city, with 1809 participants across all locations. Stakeholders included representatives from the district health office, social welfare office, local police, outreach nongovernmental organizations (NGOs), and community representatives. During the workshops, working teams were formed in each district and city, consisting of a supervisor from the health office, field officers from health offices and health centers, local NGOs, and a data manager from the health office. The workshops produced a list of hotspots, incorporating insights from stakeholders about key individuals and peak activity times at each identified location.

After the workshop phase, field officers—1 individual each from the health office, local NGO, and health center—received training on the technical implementation of mapping before visiting the hotspots. The training was conducted in groups of districts, which was a cost-effective approach to maximizing the number of trained officers. Topics covered included how to perform mapping and observations at hotspots, conduct interviews, and fill out data using paper-based forms.

The mapping team carried out hotspot observations in the next phase of the project. The team members visited specific locations known as gathering points for KPs during peak hours from July to October 2022. For quality control purposes, 1822 (10.74%) of the 16,964 observed hotspots underwent a second observation. Trained enumerators conducted interviews at these hotspots using predetermined paper-based forms to collect data. The interviews addressed various aspects, such as the number of KPs present and how often they visited the hotspots within a single day. Before the interviews, all participants were asked for their informed consent to participate in the mapping initiative. Informant interviews were conducted at each hotspot with at least 2 individuals from KPs and 2 individuals from outside KPs, such as clients of sex workers or other relevant visitors.

On the basis of the information provided by the informants, the working teams from the districts and the mapping teams assessed the reliability or confidence level of the information obtained, categorizing it as low or high. Only information rated with a high confidence level was used for further analysis. The validation criteria included complete visit times and fully completed forms. These data were subsequently used to calculate the counts of KPs per hotspot and for aggregating data at the district level.

### Community Participation

Involving community members in the field team was crucial for observing KPs in hotspots and gaining the trust of key individuals within these populations. To enhance the accuracy of the geomapping results, particularly for MSM and transgender women, district or city teams predominantly included members from MSM or transgender women communities in their field teams. A mixed approach was adopted for mapping FSWs, combining members from the FSW community with others who had experience with and knowledge about FSWs. When conducting mapping for people who inject drugs, the field team collaborated with the individuals responsible for accurately recording information on hotspot datasheets. This extensive engagement with individuals directly and indirectly involved with the KPs provided a comprehensive and nuanced understanding of the hotspot dynamics, which was crucial for developing effective HIV prevention and care strategies.

### Data Management and Analysis

The mapping project used Microsoft Excel as a data management platform. This application allowed the mapping team to input, organize, and analyze information regarding observation times, hotspot characteristics, and informant details.

A careful mathematical and statistical approach was essential for accurately aggregating the number of KPs across districts, cities, and hotspots. By using a formula based on the average number of hotspots visited in the last 24 hours, we were able to estimate KP mapping results on a larger scale. This method ensured high accuracy and minimized the risk of duplication.

The aggregation process involved consolidating the mapping results from multiple hotspots into district and city totals. By focusing on the average hotspots visited, we ensured that the figures reflected the true distribution of KPs while avoiding excessive duplication. The following simple formula was used:

∑〖in district-city = (∑〖hotspot mapping〗) / (average number of hotspots visited)〗

In this context, ∑(Hotspot mapping) represents the median number of individuals identified during peak times and Saturdays from all credible informants across all hotspots within a particular district or city. The “Average number of hotspots visited” corresponds to the mean number of distinct hotspots that a member of the KP reported visiting in the past 24 hours.

This simplified formula is the initial step in quantifying the size of KPs, although it does not fully encapsulate the complexities of KP movements and interactions [10-12]. Nonetheless, it provides a valuable baseline for refining the total KP figures at the district level, aiming to minimize the potential for duplication in the mapping process.

### Ethical Considerations

The mapping initiative was approved after receiving ethics clearance from Atmajaya University (0010A/III/PPPE.PM.10.05/06/2022). Due to the legal implications associated with activities such as sex work and drug injection in Indonesia and the ongoing stigma and discrimination faced by other KPs, verbal consent was carefully obtained during the interview phase to ensure the protection of participants’ rights and confidentiality. No financial incentives were provided. The data obtained from the mapping initiative are securely stored in a password-protected folder at Udayana University and the Ministry of Health. The stored and analyzed data do not include any personally identifiable information.

## Results

### Mapping Results Based on Stakeholder-Provided Information During the District- or City-Level Workshops

Through collaborative, multisectoral hotspot identification at the district or city level workshops, 18,339 hotspots were identified nationwide, spanning 201 districts and cities. On the basis of stakeholder-provided information, it was estimated that, of these 18,339 hotspots, 8030 (43.79%) were frequented by FSWs, 6923 (37.75%) by MSM, 2718 (14.82%) by transgender women, and 668 (3.64%) by people who inject drugs.

The analysis of hotspot identification revealed a clear trend: districts and cities categorized as “comprehensive” have a significantly higher average number of hotspots for each KP group than those labeled “medium” and “basic.” Specifically, for MSM, on average, there were 48 hotspots per district or city in the comprehensive category compared to 24 in the medium category and 12 in the basic category; for transgender women, there were 18 hotspots on average in the comprehensive category, 11 in the medium category, and 7 in the basic category; FSWs reported an average of 54 hotspots in the comprehensive category, 30 in the medium category, and 16 in the basic category; while people who inject drugs had the fewest hotspots, averaging 12 in the comprehensive category, 6 in the medium category, and 1 in the basic category.

While the types of hotspots are often similar across districts with comprehensive, medium, and basic classifications for each KP, preferences for specific hotspot types varied among the different groups (Table 1). For MSM and people who inject drugs, open spaces were the predominant hotspot type at all priority levels of districts and cities, with the highest proportions in the comprehensive category. By contrast, salons were the primary hotspot type for transgender women, especially in the comprehensive and medium districts. Although slightly less prevalent in basic districts, salons remained a significant hotspot type. Entertainment venues consistently emerged as the most common hotspot for the FSW population across all categories, underscoring their role as central activity points for FSWs.

**Figure 1:**
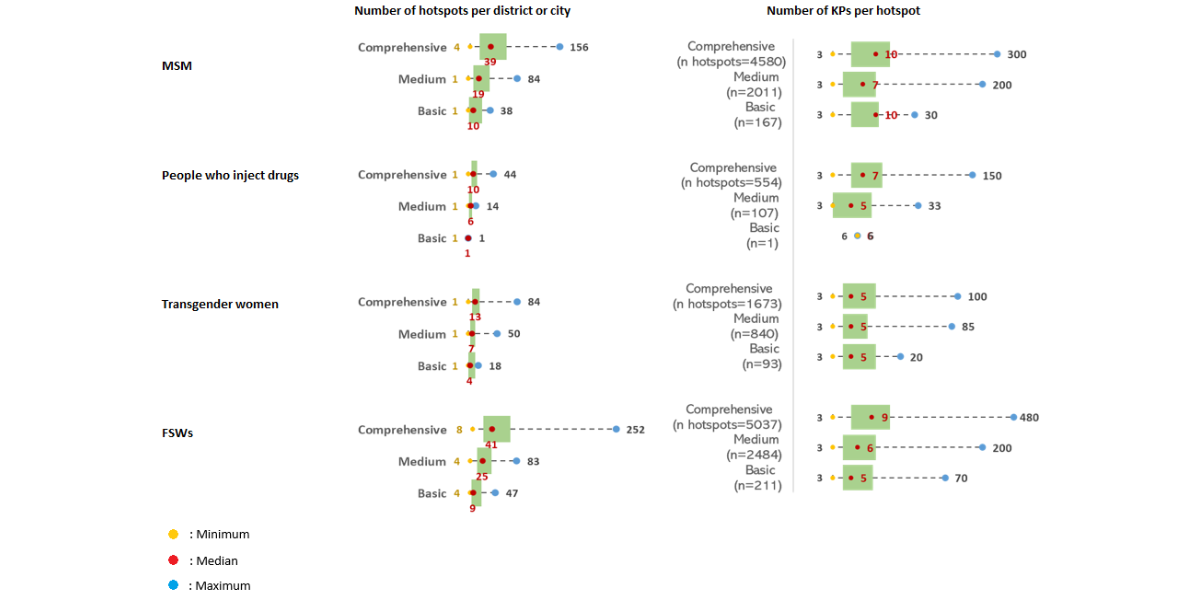
Differences in the number of key populations (KPs) identified by stakeholders across the priority categories of districts and cities. FSW: female sex worker; MSM: men who have sex with men.

Overall, mapping the number of KPs at the district or city level, based on stakeholder-provided information, revealed broader disparities between the priority categories of districts and cities compared to the individual hotspot levels. These variations are depicted in Figure 1, illustrating the differences in the number of hotspots and KP numbers across the priority categories of districts and cities.

**Table 1:** Types of hotspots identified by the stakeholders (n=17,092).

Types of hotspots	MSM^a^ (n=6851), n (%)				People who inject drugs (n=651), n (%)				Transgender women (n=2687), n (%)				FSWs^b^ (n=6903), n (%)		
	Comprehensive (n=97)	Medium (n=89)	Basic (n=15)	Comprehensive (n=97)		Medium (n=89)	Basic (n=15)	Comprehensive (n=97)		Medium (n=89)	Basic (n=15)	Comprehensive (n=97)		Medium (n=89)	Basic (n=15)
Open spaces	1178 (26)	562 (27)	49 (28)	336 (60)		62 (58)	1 (100)	353 (21)		169 (19)	20 (19)	562 (11)		276 (11)	43 (18)
Cafes	1127 (24)	529 (26)	53 (30)	42 (8)		6 (6)	—^c^	61 (4)		73 (8)	21 (20)	753 (15)		636 (25)	75 (31)
Recreation areas	1038 (23)	433 (21)	26 (15)	119 (21)		30 (28)	—	91 (5)		53 (6)	5 (5)	225 (4)		173 (7)	16 (7)
Salons	437 (9)	257 (12)	34 (19)	1 (0.2)		1 (1)	—	819 (48)		488 (55)	46 (45)	116 (2)		94 (4)	10 (4)
Entertainment venues	278 (6)	92 (4)	07 (4)	6 (1.1)			—	44 (3)		33 (4)	6 (6)	1,180 (23)		526 (21)	45 (19)
Residences	449 (10)	132 (6)	04 (2)	37 (7)		5 (5)	—	248 (15)		30 (3)	1 (1)	524 (10)		298 (12)	6 (2)
Fixed places	—	—	—	—		—	—	48 (3)		33 (4)	1 (1)	753 (15)		238 (9)	12 (5)
Massage parlors	23 (0.5)	8 (0.4)	1 (0.6)	—		—	—	2 (0.1)		2 (0.2)	—	707 (13.7)		159 (6.3)	14 (5.8)
Hotels	61 (1)	38 (2)	4 (2)	2 (0.4)		—	—	14 (0.8)		8 (0.9)	1 (1)	321 (6.2)		108 (4.3)	20 (8.3)
Malls or markets	6 (0.1)	3 (0.1)	—	—		—	—	—		0 (0)	—	—		7 (0.3)	—
Others	14 (0.3)	8 (0.4)	—	16 (3)		3 (3)	—	13 (0.8)		2 (0.2)	2 (2)	6 (0.1)		—	—

^a^MSM: men who have sex with men.

^b^FSW: female sex worker.

^c^Not applicable.

### Mapping Results Based on Observations and Interviews

Of the 18,339 hotspots identified across the 201 districts and cities, 16,964 (92.5%) were successfully observed. Despite these efforts, some of the hotspots remained unobserved: 6.4% (443/6923) of the MSM hotspots, 6.9% (46/668) of the hotspots frequented by people who inject drugs, 8.64% (235/2718) of the hotspots frequented by transgender women, and 8.1% (651/8032) of the FSW hotspots.

The primary reason for not observing some hotspots was changes in the field, as reported by the informants. Notably, several open spaces, previously gathering places for KPs, were no longer used. Other hotspot types, including eateries, entertainment venues, recreation areas, salons, fixed locations for selling sex, massage parlors, hotels, and malls or markets, were found to be closed or no longer operational. A few hotspots remained unobserved due to geographic challenges, where the distance from the district or city capital posed a barrier for the mapping officers.

The majority of the hotspot observations (13,832/16,964, 81.53%) for the 4 KPs were conducted from the afternoon into the evening, specifically between noon and 11:59 PM. The most active observation period was noted to be between noon and 5:59 PM, accounting for 46% (7803/16,964) of all observations across the KPs, indicating this as the prime time for data collection. Nighttime observations (6 PM- 11:59 PM) held particular importance for transgender women and FSWs, with nearly one-third (770/2483, 31.01%) to more than one-third (2730/7379, 37%) of their total observations commencing during these hours. Conversely, morning observations (6 AM-11:59 AM) were less frequent across all populations, with people who inject drugs mainly showing the lowest rate of observations during this time period.

**Figure 2:**
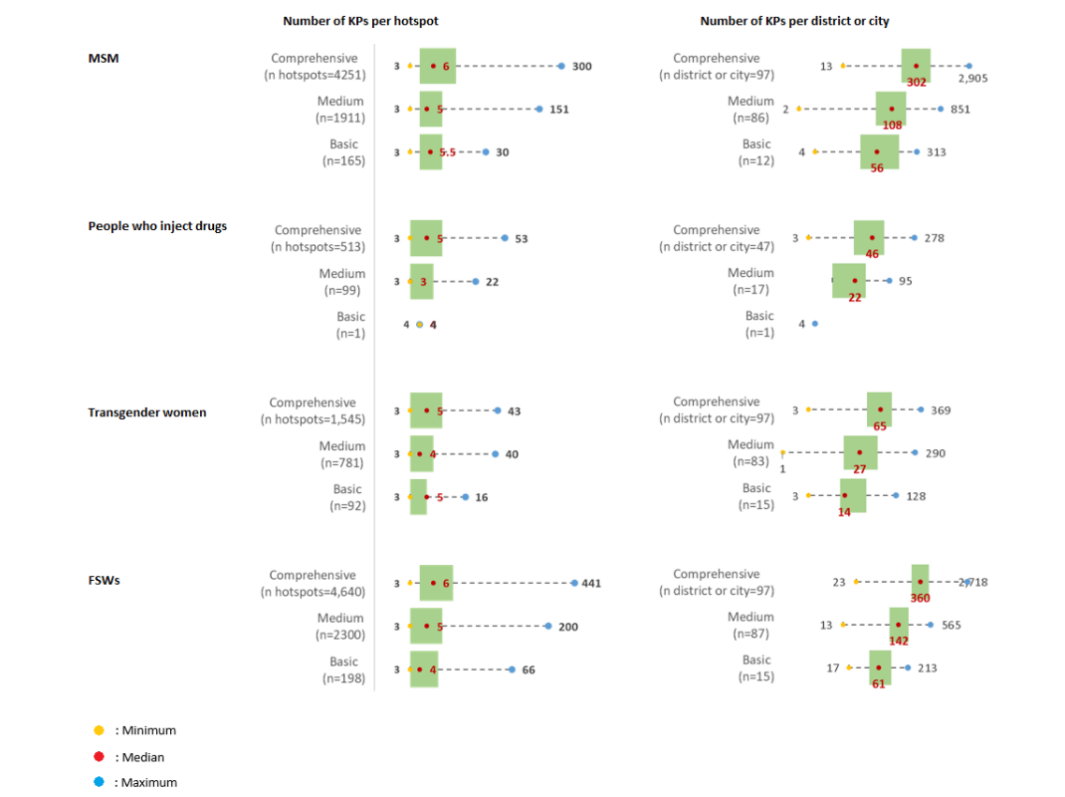
Distribution of the number of key populations (KPs) observed per hotspot and per district or city. FSW: female sex worker; MSM: men who have sex with men.

The observed data revealed a pattern in the distribution of KP numbers, which generally corresponded with the estimates provided by stakeholders at the district or city level, albeit with a slightly lower median value. This consistency suggested that, despite the lower observed figures, the overall trends and relative differences among districts and cities of varying priority categories still mirrored the stakeholder estimates. The observations verified significant concentrations of KPs at certain hotspots. Nonetheless, the maximum values recorded indicate that while some hotspots were highly populated, their population densities were typically not as high as initially projected by the stakeholders. Figure 2 graphically displays these findings, illustrating the number of KPs per hotspot in different district or city categories.

Furthermore, the mapping results incorporate data from interviews conducted with the informants during the observations. During the hotspot observation period, the mapping team interviewed 49,346 informants, averaging 2 to 3 informants per hotspot. Of these 49,346 informants, 31,588 (64.01%) were members of KPs, while the remaining 17,758 (35.99%) were not classified as KPs. Specifically, on average, 2.2 informants from among people who inject drugs were interviewed per hotspot (1346 informants in total). In hotspots frequented by MSM and transgender women, on average, 1.9 informants were interviewed per hotspot (12,415 MSM and 4699 transgender women informants in total). In hotspots frequented by FSWs, on average, 1.8 informants were interviewed per hotspot (13,128 FSWs in total).

These data are categorized into 3 groups: hotspot conditions during peak times, hotspot conditions on Saturdays, and hotspot conditions before the COVID-19 pandemic. The aggregated mapping, displaying the distribution of KPs at hotspots during peak times across different districts and cities, revealed a widening gap between districts and cities categorized as comprehensive and those classified as medium or basic. This disparity is attributed to the higher number of hotspots in comprehensive districts and cities, which amplifies the difference in the mapped presence of KPs at these hotspots.

When the mapping of KP numbers at hotspots during peak times is compared with the stakeholders’ estimates from Figure 1, it becomes evident that the mapped numbers during peak times are generally lower than the estimates provided by stakeholders. In addition, a comparison of the mapping distribution of KP numbers at peak times with previous data shows a typically lower Saturday attendance.

Using information from informants about the population density at hotspots before the pandemic compared to the current situation, it was found that 63.12% (10,708/16,964) of the hotspots had at least 1 informant who provided data on the number of KPs at peak times before the COVID-19 pandemic. The hotspots with the most prepandemic population data were FSW hotspots (2594/7379, 35.15%), followed by hotspots frequented by transgender women (677/2483, 27.27%) and hotspots frequented by people who inject drugs (129/622, 20.7%). Only 1 of the 6480 MSM hotspots lacked KP count information for peak times before the COVID-19 pandemic.

The analysis results regarding the median difference in the number of KPs at hotspots are detailed in Table 2. These results show a median decrease in the number of KPs present in almost all hotspot types at the time the study was conducted compared to the prepandemic period. For the MSM population, open spaces, cafes or stalls, and recreation or socialization areas exhibited a noticeable median decline: open spaces dropped from 15 (IQR 8-25) to 10 (IQR 7-20), cafes or stalls from 13 (IQR 8-20) to 10 (IQR 7-12), and recreation or socialization places from 15 (IQR 10-25) to 10 (IQR 7-20). The population of people who inject drugs also saw a decline, particularly in entertainment venues, with the prepandemic median of 15 (IQR 8.5-22.5) decreasing to 7.5 (IQR 6.25-8.5) at the time of the study was conducted.

Transgender women experienced a median decline in attendance across all hotspot types, with the most substantial decreases observed in massage parlors (from 15 to 8.5) and sex-selling locations (from 10 to 7.5). Similarly, FSW hotspots exhibited a median decline in attendance in most locations, with the most significant drops in entertainment venues (from 15 to 11) and massage parlors (from 8 to 6).

**Figure 3:**
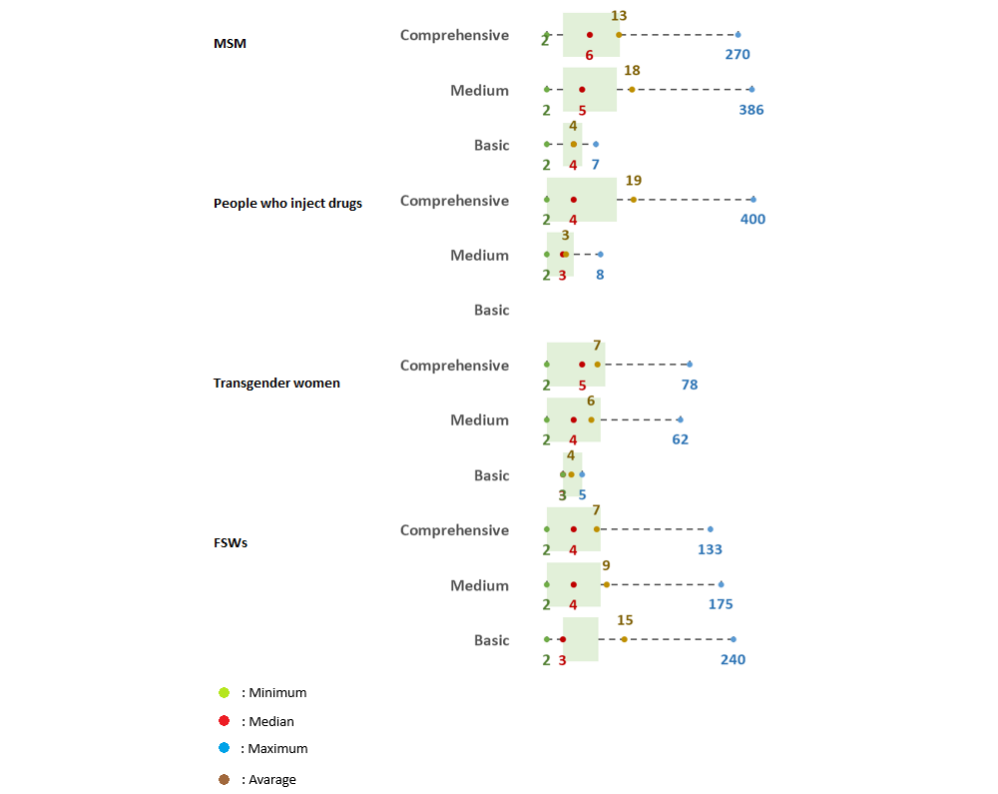
Distribution of key populations who never visited hotspots, based on mapping data. FSW: female sex worker; MSM: men who have sex with men.

Finally, interviews with informants were conducted at these hotspot locations to account for KPs who did not frequent hotspots in our population estimates (Figure 3). For the MSM population in comprehensive districts and cities, the median number of individuals who had never visited a hotspot was 13, with a maximum of 270. In the context of the population of people who inject drugs within comprehensive districts and cities, the median was 4, escalating to a maximum of 400. This indicates that many people who inject drugs may not interact with existing hotspots. The median in comprehensive districts and cities for transgender women was 6, peaking at 78. Conversely, the FSW population in comprehensive districts displayed a median of 7, with the highest value recorded being 133.

**Table 2:** Distribution of key population numbers at hotspots before and during mapping (after the COVID-19 pandemic), categorized by hotspot type and key population.

Hotspot types	MSM^a^, median (IQR)			People who inject drugs, median (IQR)			Transgender women, median (IQR)			FSWs^b^, median (IQR)	
	Before the pandemic	During mapping	Before the pandemic		During mapping	Before the pandemic		During mapping	Before the pandemic		During mapping
Open spaces	15 (8-25)^c^	10 (7-20)^d^	8 (5-15)^c^		7 (5-13.75)^d^	10 (7-15)^c^		10 (6-13)^d^	12 (7-20)^c^		10 (6-15)^d^
Cafes	13 (8-20)^c^	10 (7-17)^d^	6 (4-10)^c^		5 (4.25-9.75)^d^	8.5 (5-15)^c^		6 (4-10)^d^	10 (6-17)^c^		8 (5-13)^d^
Salons	10 (7-15) ^c^	8 (5-13)^d^	9 (9-9)^d^		12 (12-12)^c^	8 (5-12)^c^		6 (4-10)^d^	7 (5-11)^c^		5 (4-8)^d^
Entertainment venues	12 (8-20)^c^	10 (6-18.25)^d^	15 (8.5-22.5)^c^		7.5 (6.25-8.5)^d^	10 (6-13)^c^		6 (4-10.5)^d^	15 (7-28)^c^		11 (6-21)^d^
Recreation areas	15 (10-25)^c^	10 (7-20)^d^	9 (5-15)^c^		8 (5-15)^d^	10 (6-15)^c^		7.5 (5-12.75)^d^	9 (6-15)^c^		7 (5-10)^d^
Residences	12 (7-20)^c^	10 (6-15)^d^	10 (7-12.5)^c^		10 (5.5-12)^d^	10 (6-15)^c^		7 (5-10)^d^	12 (6-20)^c^		10 (6-15)^d^
Fixed places	—^e^	—	—		—	10 (7.75-15)^c^		7.5 (6-10)^d^	11 (6-25)^c^		9 (5-20)^d^
Massage parlors	14 (7.5-20)^c^	10 (6-15.5)^d^	—		—	15 (12.5-7.5)^c^		8.5 (7.75-9.25)^d^	8 (5-15)^c^		6 (4-12)^d^
Hotels	8 (5-20)^c^	9 (5-15)^d^	4 (4-4)^d^		5 (5-5)^c^	9.5 (5.75-15)^c^		8 (5.75-10)^d^	13 (8-20)^c^		10 (6-15)^d^
Malls or markets	7.5 (4.25-13.75)^c^	5.5 (4-13)^d^	—		—	—		—	5 (5-6)^c^		4 (3-6)^d^
Others	7 (6-8)^c^	6 (5-7)^d^	10 (4.75-25)^c^		5.5 (4-21.25)^d^	15 (9-22.5)^c^		12 (4.5-13.5)^d^	20 (10-20)^c^		10 (10-13)^d^

^a^MSM: men who have sex with men.

^b^FSW: female sex worker.

^c^Higher.

^d^Lower.

^e^Not applicable.

### Aggregation of Mapping Results in Districts and Cities

**Figure 4:**
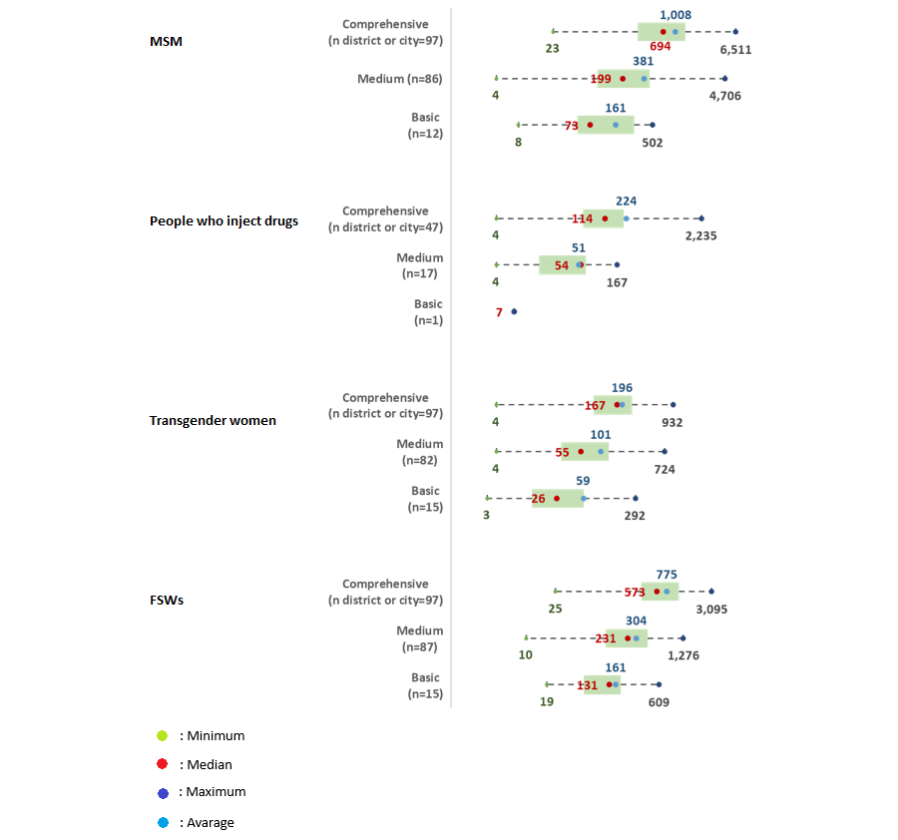
Distribution of aggregated mapping results across districts and cities, reported by key population type. FSW: female sex worker; MSM: men who have sex with men.

An aggregation of the mapping results was performed using the data collected from observations and interviews. The aggregations were carried out for each KP and district or city classification. Figure 4 illustrates the distribution of these aggregated mapping results across districts and cities, categorized by KPs and district or city classifications. The average number of mapping results per district or city for the MSM population in the comprehensive category was 1008 (median 694; range 381-6511). The wide range indicates a significant variation in mapping results across districts or cities in this category. By contrast, the medium category showed an average of 381 (median 199) mapping results. The basic category presented even lower figures, with an average of 161 (median 73), exhibiting more limited variation.

The population of people who inject drugs exhibits a similar trend but with overall lower numbers. The average aggregated mapping results in comprehensive districts and cities was 224 (median 114). The averages in the medium and basic categories were 51 (median 54) and 7, respectively, highlighting this population’s disparity in aggregated mapping data.

For transgender women, the comprehensive category had an average of 196 (median 167) mapping results. In the medium category, the average decreased to 101 (median 55). The basic category showed an even lower average of 59 (median 26). Regarding the FSW population, comprehensive districts and cities averaged 775 (median 573) mapping results. The medium and basic categories saw a decrease in averages, dropping to 304 (median 231) and 161 (median 131), respectively.

Subsequently, the mapping results were compared with data from ongoing outreach programs. Table 3 provides the median number of populations mapped successfully and compares these figures with the outreach data. Generally, the mapping results yielded lower numbers than the outreach results collected throughout the program, and there was a notable discrepancy between the estimated numbers of MSM through mapping exercises and the coverage of HIV outreach and testing programs. These estimates indicated that the actual number of MSM was >50% lower than the program coverage. A similar disparity existed for FSWs, with estimates being 13% lower than outreach results. By contrast, for people who inject drugs and transgender women, the estimated numbers exceeded the extent of existing programs. Notably, the estimated figures for people who inject drugs were twice as high as the program coverage.

**Table 3:** Comparison of distribution of key population (KP) mapping data with program data by district or city.

KPs		Informed estimates	Observed	Saturdays	Peak times	Empirical estimates	Coverage by ORWs^a^	Had HIV test
**MSM^b^**								
	Values, median (IQR; range)	238 (105.5-560; 2-4481)	189 (78-384; 2-2905)	275 (109-553; 4-4930)	323 (119-601; 4-5500)	376 (152-822; 4-6511)	2218 (1240-3412106-14,996)	934 (239-1899.5; 2-11,472)
	Values, mean (SD)	425 (556)	299 (380)	470 (617)	499 (651)	697 (919)	2778 (2360)	1338 (1580)
**People who inject drugs**								
	Values, median (IQR; range)	55 (21.5-119; 2-535)	36 (17-68; 2-278)	55 (19-97; 2-560)	64 (31-131; 2-555)	85 (36-179; 4-2235)	223.5 (80.25-441.5; 3-1473)	29.5 (24.75-167; 2-1046)
	Values, mean (SD)	97 (120)	53 (59)	85 (103)	98 (108)	175 (364)	310 (323)	123 (191)
**Transgender women**								
	Values, median (IQR; range)	63.5 (29-132.25; 1-717)	46 (22-871-369)	63 (31.75-143.5; 3-496)	73 (31-155; 3-507)	98.5 (38.75-208.5; 3-932)	158.5 (67-266.25; 14-814)	59 (16-167.5; 1-723)
	Values, mean (SD)	95 (97)	63 (62)	100 (101)	106 (101)	148 (154)	194 (155)	102 (119)
**FSWs^c^**								
	Values, median (IQR; range)	309 (143-538; 22-3603)	211 (119-371; 13-2718)	309 (150-536; 10-3087)	329 (175-551; 19-3099)	364 (180-660; 10-3095)	707 (309-1435; 69-3560)	291 (68.25-592.25; 1-2670)
	Values, mean (SD)	465 (573)	317 (357)	436 (468)	465 (481)	528 (515)	956 (774)	431 (480)

^a^ORW: outreach worker.

^b^MSM: men who have sex with men.

^c^FSW: female sex worker.

## Discussion

### Principal Findings

The findings from district and city workshops showed that comprehensive districts and cities generally had significantly higher hotspot locations than medium and basic areas; for example, the average number of MSM hotspots per district or city in the comprehensive category was 48. This figure contrasts with 24 in the medium category and 12 in the basic category. It is important to target specific areas for HIV and AIDS interventions, such as open spaces for MSM, salons for transgender women, and entertainment venues for FSWs. Although there has been a decrease in hotspot density after the pandemic, the types of hotspots identified remain consistent with those commonly recognized. The preferences of KPs for these hotspots have also shown little change. Similar trends were observed in other mapping studies conducted in different geographic settings [13,14]. Despite varying proportions among district categories, these hotspot types are critical points for intervention across all levels of district priority.

Insights from observation data show that the concentration of KPs in hotspots varied, based on the district or city category; for instance, in the comprehensive category, the median number of MSM per district or city was 302, while for people who inject drugs, transgender women, and FSWs, the median numbers were 46, 65, and 360, respectively. The comprehensive category had a significantly higher concentration of KPs per hotspot than the medium and basic categories. However, these numbers were lower than the estimates from workshop results, where the comprehensive category had higher medians: 422 for MSM, 75 for people who inject drugs, 100 for transgender women, and 471 for FSWs. This indicates a greater focus on comprehensive areas for HIV and AIDS control in Indonesia, with some attention also given to medium category areas. It is worth noting that basic areas also receive attention, including through the Global Fund Program, although this does not imply the absence of HIV prevention programs in these areas. Standard HIV testing and treatment services are available in basic areas, following the concept of allocating interventions based on geographic epidemiological conditions [15]. Moreover, key findings from field observations indicated significant fluctuations in the presence of KPs in hotspots, particularly during peak periods. This highlights the necessity for careful interpretation of the data and underscores the complexity of addressing HIV and AIDS in varied district or city contexts [16].

The comparison between workshops’ estimates of KP numbers and informant interview results revealed a consistent trend: stakeholder estimates were generally higher. This discrepancy likely stems from stakeholders basing their numbers on experience or expectations, whereas informant interviews provide a more realistic, albeit lower, representation of the actual situation. In addition, the variance might be attributed to the limited duration of observations, which often spanned only a few hours and may not have captured the full range of KP arrivals. This suggests that extending observation periods and broadening the time frame could yield a more comprehensive and accurate portrayal of KP activities [17].

The report highlights the significant impact of the COVID-19 pandemic on the mobility of KPs. There has been a decrease in the number of KPs in almost all types of hotspots compared to the prepandemic period. For the MSM population, there has been a noticeable median decline in attendance in open spaces (from 15 to 10), cafes or stalls (from 13 to 10), and recreation or socialization areas (from 15 to 10). The population of people who inject drugs also experienced a decline in attendance, particularly in entertainment venues, with the prepandemic median of 15 decreasing to 7.5 today. This aligns with academic research that documents the pandemic’s effects on mobility and social interactions, especially among KPs of individuals living with HIV [6,7,18]. Studies worldwide indicate a postpandemic decline in public space social interactions [19-21]. Understanding these dynamics is crucial for developing effective postpandemic public health interventions, particularly restoring social interactions in hotspots and preventing disease spread. Strategies should evolve in response to the altered social and epidemiological landscapes, drawing on pandemic experiences to ensure that HIV prevention and response are effectively tailored to the new context.

A comparison of the mapping results for KP distribution with the coverage of HIV prevention and testing programs revealed notable discrepancies. The actual number of MSM was >50% lower than the program coverage. A similar disparity existed for FSWs, with estimates being 13% lower than outreach results. Mapping, which uses various methods such as stakeholder inputs and direct observations, often yields lower estimates compared to the broader scope of ongoing, sustainable programs; for instance, the median number of MSM per district identified through mapping was 238, significantly lower than the median recorded by outreach program coverage, which was 2218. Similar patterns were observed for people who inject drugs, transgender women, and FSWs, where the figures from HIV outreach and testing programs consistently exceeded those from mapping exercises. This suggests that mapping provides a temporal “snapshot” and might not fully capture the year-round dynamics of KPs [11]. Therefore, integrating mapping results with data from year-long programs is crucial to gaining a more comprehensive understanding of the needs and effectiveness of HIV intervention programs [14].

Finally, the mapping process conducted across 201 Indonesian districts and cities identified several limitations that are important to acknowledge for accurate analysis and future planning. These include incomplete hotspot identification and focusing only on certain segments of KPs, leading to incomplete representation. The mapping primarily accounts for visible KP members at hotspots during observation times, omitting those who might visit at other times. Another limitation is the heavy reliance on key informants, whose reports may be affected by memory bias and challenges in estimating precise numbers, potentially compromising data accuracy.

In addition, as highlighted previously, data collection was predominantly conducted in a single visit, which fails to consider temporal changes and affects the reliability of the findings. Focusing on high or peak periods could also lead to overestimations that do not represent typical daily conditions. Consequently, these factors create challenges in extrapolating the observed “snapshot” into a more extensive understanding of KP presence over extended periods. Recognizing these limitations also presents opportunities for future refinement of mapping methodologies and strategies. Further research is essential to validate and expand upon these findings. Longitudinal data collection would be particularly beneficial, providing a deeper understanding of the evolving behaviors of KPs over time and in response to various influences, including global pandemics. Despite its limitations, the mapping exercise has yielded valuable insights into the distribution of KPs based on district or city categories, which are instrumental in enhancing HIV control efforts.

### Conclusions

In conclusion, the findings from this comprehensive mapping of KPs of people living with HIV across Indonesian districts and cities carry significant public health implications. First, identifying high-risk hotspots and their concentration in comprehensive districts highlights the urgent need for geographically targeted interventions, which can optimize resource allocation and enhance the effectiveness of HIV prevention efforts. Despite the postpandemic changes, the consistency in the hotspots frequented by KPs indicates stable behavioral patterns, which should inform the design of tailored intervention strategies. The discrepancies between stakeholder estimates and direct observations underscore the importance of robust, longitudinal data collection to understand KP dynamics better. In addition, the impact of the COVID-19 pandemic on mobility and social interactions among KPs necessitates the adaptation of intervention strategies to the new social and epidemiological realities. Addressing these challenges through improved mapping methodologies and sustained public health initiatives can significantly enhance HIV control efforts, reduce transmission rates, and ultimately improve health outcomes for KPs in Indonesia.

## Abbreviations

ART: antiretroviral therapy
FSW: female sex worker
KP: key population
MSM: men who have sex with men
NGO: nongovernmental organization
PLACE: Priorities for Local AIDS Control Efforts

## Data Availability

Data sharing is available upon request to the Ministry of Health of Indonesia. The authors do not possess the data; moreover, they are not authorized to share any data with other researchers.
